# Novel Meniscus Transfer Technique: A Case Report

**DOI:** 10.7759/cureus.63677

**Published:** 2024-07-02

**Authors:** David Yatsonsky, Jenna L Gunn, Tony Dong, Aidan Maxwell, David Sohn

**Affiliations:** 1 Orthopedic Surgery, The University of Toledo College of Medicine and Life Sciences, Toledo, USA; 2 College of Medicine, The University of Toledo College of Medicine and Life Sciences, Toledo, USA

**Keywords:** meniscus transplant, lateral meniscus injury, novel surgical technique, meniscus allograft transplantation, meniscus tear

## Abstract

The meniscus is an essential component of the knee joint, acting as a shock absorber as well as assisting in the transmission of forces. Due to the meniscus importance of the knee, much of the current literature focuses on treatment techniques that can spare and repair the meniscus when it is torn. The unique vasculature of the meniscus often makes repair difficult or, in many cases, impossible. A current focus within orthopedics has been on meniscal allograft transplantation to fill this gap. The lack of a universal surgical technique for graft fixation, along with the current failure rates, demonstrates the need for further improvements. The senior author proposes a novel technique for meniscal allograft transplantation that has shown decreased blood loss and surgical time, while also reducing intra-operative trauma to the knee.

This case reports a 16-year-old patient who underwent a right lateral meniscal allograft transplant following a large segmental defect tear of the lateral meniscus. The patient initially underwent arthroscopy and meniscectomy with screw fixation of the lateral femoral condyle lesion. After physical therapy, the patient experienced increased pain and swelling, with magnetic resonance imaging (MRI) demonstrating a meniscal defect unamenable to repair. The patient met indications for meniscal allograft transplantation given the failed meniscectomy, absence of cartilage loss and significant osteoarthritis, and the patient's age of less than 50 years old.

## Introduction

The knee is a hinge joint made up of the femur, tibia, and patellar bone. Within the knee, lying between the femoral condyles and tibial plateau, are two fibrocartilage structures known as “menisci.” The menisci act as shock absorbers and aid in the transmission of axial loads and shearing forces evenly across the knee, provide stability, and aid in lubrication of the knee joint [1]. The medial meniscus has a “U-shape” while the lateral meniscus has a “C-shape” [2]. Both are made up of fibroelastic cartilage composed of primarily water and collagen (90% type 1) [2]. Collagen fibers within the menisci run radially and longitudinally, which allow for proper expansion under compressive forces [2]. 

Nearly 60 out of 100,000 persons are estimated to experience a meniscal tear per year, with this incidence increasing [3]. This number is expected to be underestimated, given the number of people with symptomatic and asymptomatic meniscal tears that are never definitively diagnosed. Studies have found that 75% of people with osteoarthritis have meniscal tears [4]. Meniscal tears can be classified by their tearing patterns. These include vertical longitudinal, oblique, circumferential, complex, transverse or radial, and horizontal cleavage tears [5]. Degenerative complex tears of the posterior horn are most common in the elderly and often non-amenable to surgical intervention, while posteromedial compartment radial tears are the most prevalent in the general population [5]. Blood supply to each meniscus is supplied first to the periphery and originates from the lateral and medial inferior geniculate arteries [6]. This peripheral area makes up approximately 25% of the outer portion and is termed the “red zone” due to its vasculature and healing potential if conservatively treated or surgically repaired. The “white zone” is the inner 75% portion of each meniscus, which is largely avascular and has minimal potential for healing. By adulthood, only 10-30% of the peripheral menisci still have active blood supply [7]. There is a red-white zone that makes up overlapping areas between the two zones. The location of a meniscal tear is important as it helps surgeons form a proper treatment plan for patients. 

The case presented is a 16-year-old male who underwent a right lateral meniscal allograft transplant (MAT) using the senior author's novel technique. After an initial meniscectomy, the patient had increased pain and swelling, affecting daily functions such as standing and walking. 

## Case presentation

The patient is a 16-year-old male who underwent a right lateral meniscal allograft transplant using the senior author’s novel technique. The patient was playing high school football and endured an injury to the right knee. He sustained a large segmental defect tear of the lateral meniscus. He concomitantly had an osteochondral defect of the lateral femoral condyle (LFC). The patient underwent arthroscopy and meniscectomy with screw fixation of the LFC lesion. He underwent physical therapy (PT) and had increased swelling, pain, and worsening symptoms, so magnetic resonance imaging (MRI) was ordered. This imaging demonstrated his meniscal defect, which was too large to repair. He had ongoing pain affecting daily functions like standing and walking. The patient met indications for meniscal allograft transplantation given his failed meniscectomy, absence of cartilage loss and significant osteoarthritis, and his age of less than 50 years old. 

At this point, a lateral meniscal allograft transplantation was scheduled. Appropriate size-matching X-rays and MRIs were performed. The patient understood that this procedure was not aimed to return him to sports but meant to aid with goals of functional daily living. The lateral meniscus allograft transplant was performed on 04/20/2016 utilizing the senior author’s novel technique. The patient was placed in a hinged-knee brace held in extension and advised to remain non-weight bearing (NWB) on the operated lower extremity until six weeks postoperatively and advised to take Aspirin 325 mg daily for deep vein thrombosis (DVT)/venous thromboembolism (VTE) prophylaxis. The patient experienced paresthesia in superficial and deep peroneal nerve distributions 0/5 extensor hallucis longus (EHL) strength postoperatively consistent with foot drop. Electromyography (EMG) was ordered, and the patient was given ankle foot orthosis (AFO) and aggressive physical therapy for stretching and range of motion (ROM) exercises for six weeks at two visits per week. The patient had an EMG completed, and a lesion was observed near the fibular head. It was decided to take the patient back for surgical exploration and peroneal nerve decompression on 07/21/2016. The nerve was visualized in its entirety about the fibular head with no lesions. The patient continued AFO use and PT. The patient returned on 12/13/2016. His pain and range of motion in the knee improved, and he could dorsiflex his ankle. His AFO was discontinued, and the patient was recommended to follow-up in three months. The patient continued to do well clinically and did not return to follow-up. 

**Figure 1:**
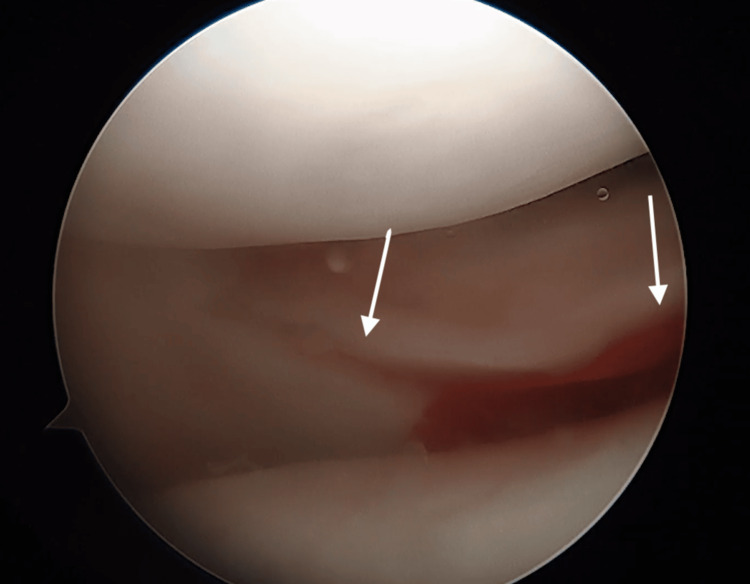
Large segmental defect of the lateral meniscus. Arthroscopic view of the large segmental defect tear of the right lateral meniscus.

**Figure 2:**
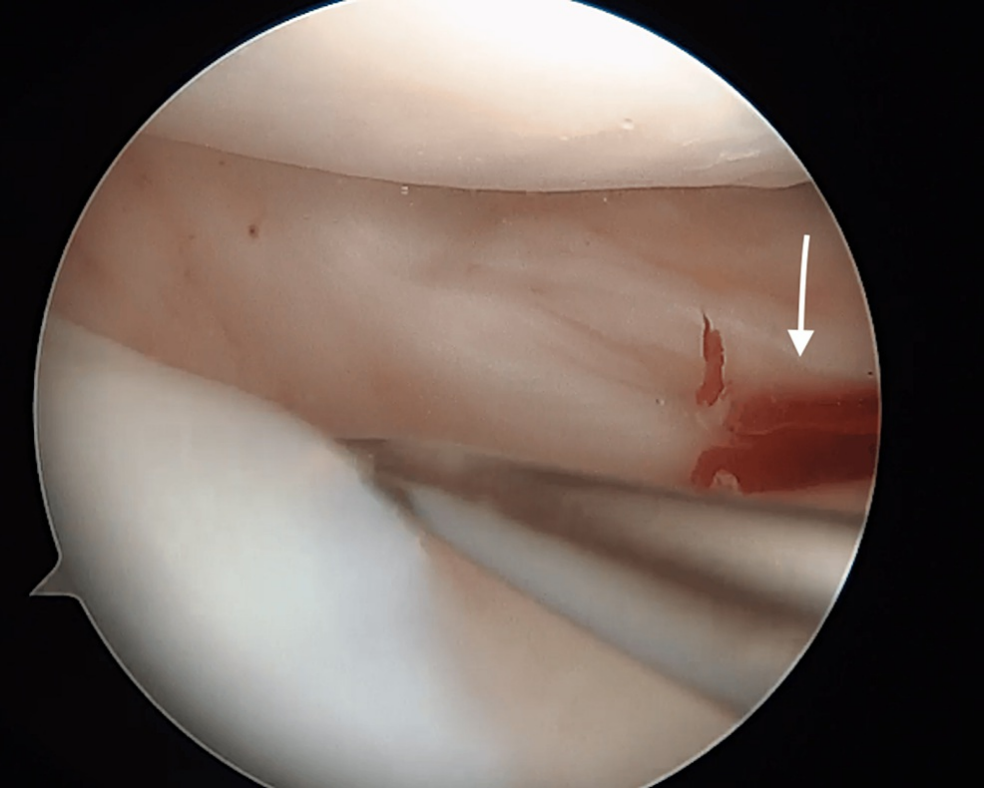
Proximal aspect of the meniscus near the posterior horn. Arthroscopic view of the proximal aspect of the meniscus near the posterior horn.

The senior author of this case has used this novel technique for select patients who were candidates for meniscal transplantation subsequently with great success; all but one who underwent this procedure did very well over the past seven years. One patient who was non-compliant with restrictions fell, tearing the transplant, and was referred to orthopedic reconstruction (Figures 1, 2). 

## Discussion

Current treatment options typically include non-operative measures, meniscectomy and debridement, surgical repair via sutures, and transplant. Non-operative measures are indicated for patients with tears in the “white zone,” older patients with degenerative tears, asymptomatic patients, those with longitudinal tears less than 1 cm in length, and stable partial tears [8].  Non-operative approaches include activity modification, nonsteroidal anti-inflammatory drugs (NSAIDs), and physical therapy. Although exercise programs through physical therapy have been shown to provide pain relief and improvements in functionality, around a third of these patients will progress to a meniscectomy or meniscal debridement [8]. Meniscectomies and debridement are indicated for patients with persistent pain and/or mechanical issues refractory to conservative treatment or from a failed meniscal repair, meniscal tears in the white zone in symptomatically active patients, and patients with coexisting degenerative and synovial fluid changes [6]. Total meniscectomy was historically the treatment for meniscal tears; however, over the years, studies have demonstrated the clear biochemical and arthritic changes that develop within the knee joint after complete removal of the menisci [5]. For this reason, the main practice is to preserve as much meniscal tissue as possible. Therefore, whenever a meniscectomy is indicated, arthroscopic partial meniscectomy or debridement is preferred. 

Historically, meniscal repairs have been reserved primarily for younger populations and performed only when the tear is in the red or red-white zone and has the potential to heal. The preferred repair technique by physician zone is via an arthroscopic, all-inside technique with the placement of vertical, non-absorbable 2-0 sutures [8]. Inside-out repair techniques remain the gold standard for bucket-handle tears of the medial and lateral meniscus, and the outside-in repair technique is commonly utilized for anterior horn tears. The all-inside technique is associated with increased costs; however, it has demonstrated decreased morbidity and lower complication rates in comparison to the inside-out technique [8]. Despite advances in meniscal repairs, 10-20% are associated with poor outcomes, and approximately 38% of these progress to failure [8]. Failed meniscal tears force surgeons to make difficult decisions-attempt a second repair, perform a partial meniscectomy to provide symptomatic relief, or attempt a meniscal transplant. However, even partial meniscectomies have demonstrated the progression of degenerative changes decades down the line [1]. Difficult decisions must also be made when deciding treatment routes for symptomatic young patients with tears in the white, avascular zone with minimal chance of healing. Due to the importance of the menisci in absorbing and distributing force and preventing arthritic and biochemical changes within the knee joint, emphasis has been placed on finding a replacement for the meniscus. Reconstruction and transplantation have been a continued focus within orthopedic research to provide a possible solution to this dilemma. 

Current meniscal allograft transplant techniques

The first free meniscal transplantation was performed in 1984 by Milachowski and Wirth [9]. There have been various types of substitutes used over the years; however, the meniscal autograft, meniscal allograft, and meniscus scaffolds are the substitutes clinically used today. Criteria for meniscal transplant indications include patients under 50-55 years old with a failed prior meniscal repair attempt, minimal joint space narrowing, and a stable, well-aligned knee [8]. Smoking, significant arthritic changes (regardless of origin) in the knee joint, and obesity are considered contraindications [10]. 

There have been several meniscal allograft transplant (MAT) techniques described, including both open and arthroscopic, as well as fixation of the transplant via a bone trough, a bone plug, or soft tissue [11]. Lateral and medial meniscal transplant techniques vary. Current meniscal transplantation methods are evolving. Allografts are typically stored as fresh frozen for prolonged preservation [12] and sterilized via a variety of techniques. Fixation of the allograft within the knee joint is done by three different methods. These include soft tissue suture fixation, bone plugs, and bone bridges [13]. Bony fixation with bone bridges and bone plugs has demonstrated superior biochemical properties with fewer postoperative complications compared to suture fixation and has become the gold standard for meniscal transplants [14]. The current postoperative course includes placing the patient in a locked hinge-knee brace and limiting movement of the operative knee from 60 degrees to full extension for the first two weeks [15]. From two weeks to four weeks postoperatively, flexion to 90 degrees is allowed with full extension. Finally, from four weeks on, a full range of motion is encouraged [15]. The first four to six weeks consist of minimal weight-bearing with crutches, with gradual progression to normal ambulation [15]. At six weeks, patients are allowed to cycle but must avoid any squatting or twisting for the first six months and refrain from playing sports for up to one year postoperatively [15]. 

Although meniscal allograft transplantation has shown promising outcomes, including a >70% graft survivorship at 10 years for isolated MAT in a recent study, as well as increased patient satisfaction during short-term follow-up [16], there are still some common complications. These complications include graft failure, extrusion, infection, arthrofibrosis, the need for reoperation, aseptic synovitis, cellulitis, hematoma, limited range of motion, joint effusion, and bone plug loosening; however, graft failure remains a main issue [17]. Graft failure remains a main issue. A 2018 meta-analysis with 1637 meniscal allograft transplants with a mean age of 34 years old showed a failure rate of 12.6%, with graft failure meaning persistent pain postoperatively, the need for complete removal of the graft, or a tear that required revision [13]. With evolving surgical techniques and differences in postoperative management, it can be difficult to draw conclusions about meniscal allograft transplantation. Although it has been proven that MAT is a safe and effective option for patients with meniscal deficiencies that meet the indicated criteria, the lack of an accepted universal surgical technique for fixation along with the current failure rates demonstrate that there is room for improvement and further research.  

Current lateral meniscus allograft transplant techniques  

The bone bridge technique is typically done for lateral meniscus transplants due to the proximity of the posterior and anterior horns of the lateral meniscus [12]. A bone bridge is created from an allograft bone block with the corresponding allograft meniscus. The bone block is cut and shaved down to fit into a pre-made bone tunnel or channel made by the surgeon between the “…anterior and posterior meniscal root attachments” [13]. 

Prior to beginning, a normal diagnostic arthroscopy is performed to ensure intact anterior cruciate ligament (ACL) and posterior cruciate ligament (PCL) structures, ensure bone integrity, and rule out any other injuries with an anterolateral and anteromedial portal site approach [15]. The lateral meniscus is debrided and shaved down, leaving only about 1-2 mm of bleeding remnant within the joint capsule [13].  The entrance for a bone channel is made via a longitudinal incision 10 mm in length through the patellar ligament [15]. Bone channel designs include trough, keyhole, dovetail, and slot [15] and are decided based upon surgeon preference. The bone channel serves as a guide for the placement of the bone block. Through this accessory incision, a notchplasty is performed with a 4 mm burr, curette, and electrocautery to create a superficial reference slot that is 4 mm in depth and line with the slope of the tibial plateau. A hooked depth gauge is placed within the superficial reference slot to measure the anteroposterior length of the tibial plateau. A guide pin is placed distal and parallel to the superficial reference slot, utilizing the hook gauge as a reference, and advanced to, but not through, the posterior cortex [15]. An 8 mm blind socket is created with an 8 mm cannulated drill bit that follows the guide pin. A “bullnosed” box chisel is placed within the blind socket, and the “roof” of the blind socket is cut off to create the bone channel measuring 8 mm wide and 10 mm deep [12,15]. This area is then smoothed out with a rasper. Next, a posterolateral incision is made on the ipsilateral side to allow for the passage of the suture. A meniscal repair cannula is passed through the contralateral arthroscopic portal site to the lateral tibial plateau with a long, nitinol suture passing wire [15]. The distal end of the wire is passed through the posterolateral incision, and the proximal end is pulled through the anterior arthrotomy incision previously made through the patellar ligament. Polydioxanone suture (PDS) traction sutures that are secured to the meniscal allograft are passed through the nitinol loop and secured [12]. Pulling of the nitinol suture passing wire allows for the passage of the bone block through the anterior arthrotomy and into the bone slot [12]. An inside-out vertical mattress suture technique is used to fixate the allograft meniscus to the surrounding capsule with 2-0 non-absorbable sutures [13]. Once the bone block is properly reduced within the bone slot and in its proper orientation within the knee joint, fixation of the bone block occurs with a “...7x23 mm bioabsorbable cortical interference screw…” [16]. All incision sites are closed, and the patient is placed in a hinged-knee brace held in extension [16].  

Current medial meniscus allograft transplant techniques 

The bone plug technique is often preferred over the bone trough technique for medial meniscus allograft transplantation due to the wider space between the anterior and posterior roots of the medial meniscus [17]. As with the lateral meniscus allograft transplantation, a diagnostic arthroscopy is first performed via anteromedial and anterolateral portal sites. The integrity of other ligamentous structures and the ruling out of chondromalacia is confirmed. The medial meniscus is similarly debrided and shaved down, leaving only about 1-2 mm of bleeding remnant within the joint capsule [13]. Bone plugs are prepared utilizing an allograft bone block with an attached medial meniscus. Anterior and posterior plugs are made approximately 7-8 mm in diameter and 8-10 mm long with care to not violate the articular surface [17]. Corresponding 1.5 mm drill holes are made within each bone plug to aid in the insertion and fixation of the allograft meniscus [17]. A non-absorbable, high-strength traction suture is passed through each of these drill holes, secured to the soft tissue, and then passed back out of the drill holes [15]. A pull-in suture is placed at the junction of the middle and posterior thirds of the meniscal soft tissue to later help with insertion [15].  

An anterior accessory incision is made at the level of the meniscal horn attachments and in line with the patellar tendon fibers to allow for the eventual passage of the graft [15]. Next, a posteromedial incision is made for visualization of the posterior horn attachment site and future retrieval of sutures during capsule repair [15]. Careful dissection past the superficial fascia and pes anserine group is done and taken down to the capsule and the medial head of the gastrocnemius [15]. A plane is made between the two, and the posterior horn attachment is then debrided [15].  Next, a guide pin is placed through the anterior accessory incision, positioned at the posterior attachment site, and an 8 mm drill is used to overream and create a posterior tibial tunnel for future placement of the posterior bone plug [15]. A looped passing suture is placed through this posterior tibial tunnel and passed out through the anterior accessory incision to help with the passage of the posterior bone plug [13]. Next, the anterior accessory incision is extended from the joint line and proximally to the patella with the knee in flexion to create a mini-arthrotomy for full exposure of the anterior horn attachment site [17]. The anterior horn attachment is exposed, and similarly, an 8 mm drill is used to create a tunnel corresponding to the size of the anterior horn bone plug. The pull-in suture initially placed in the allograft meniscus is passed through the anterior accessory incision and pulled out through a posteromedial accessory incision [13]. The posterior bone plug sutures are then passed through the looped passing suture that was previously placed in the posterior tibial tunnel [13]. The meniscal allograft is pulled into the joint with gentle traction of the allograft sutures, and the posterior plug is oriented into its corresponding tunnel and secured with a cortical button on the anteromedial tibia [13,17]. Similarly, the anterior bone plug traction sutures are passed through the anterior tibial tunnel with a Hewson suture passer [13]. These sutures aid in the reduction and proper orientation of the anterior bone plug into the anterior tibial tunnel. This bone plug is then secured with a cortical button as well [13]. The knee is placed in full extension, and the allograft meniscal soft tissue can be secured within the joint capsule using an inside-out technique with vertical mattress 2-0 braided non-absorbable sutures [13,17]. Standard closure of portal and incision sites is performed, and the patient is placed in a knee-hinge brace held in extension.

New technique 

**Figure 3:**
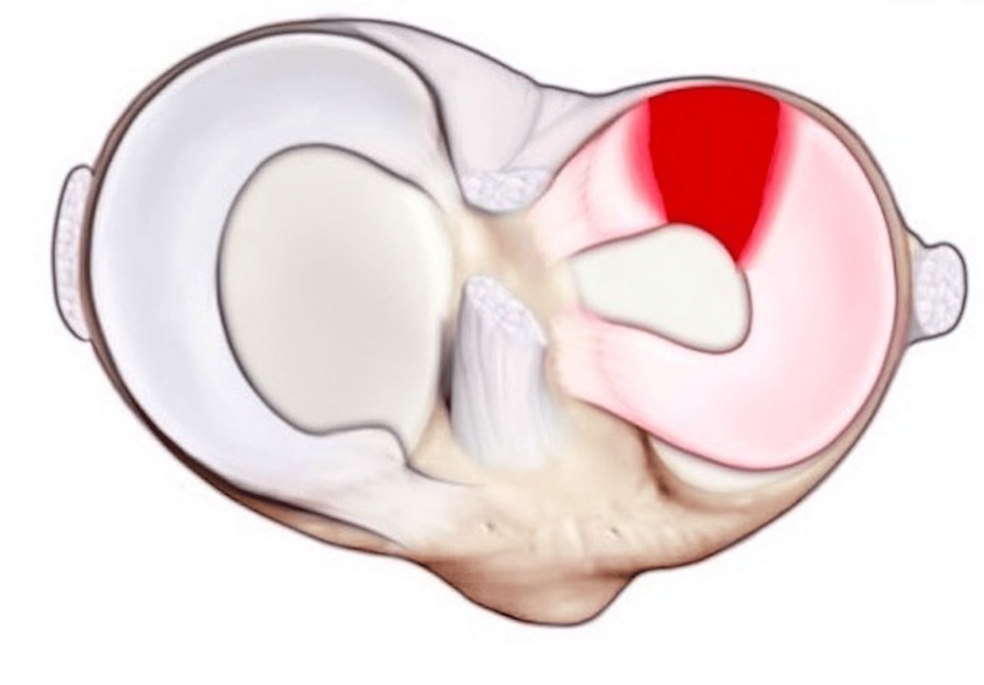
Menisci anatomy viewed in situ on the tibia. Image shows injury to the lateral meniscus.

**Figure 4:**
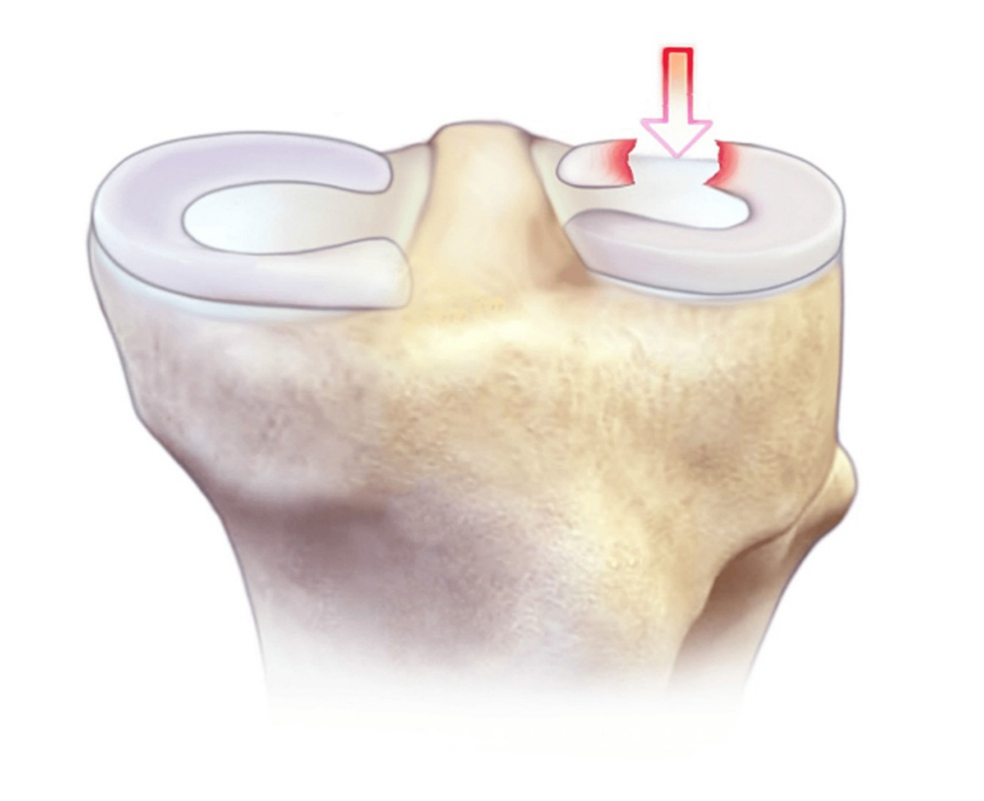
This image shows a segmented tear of the lateral meniscus prior to surgical repair.

**Figure 5:**
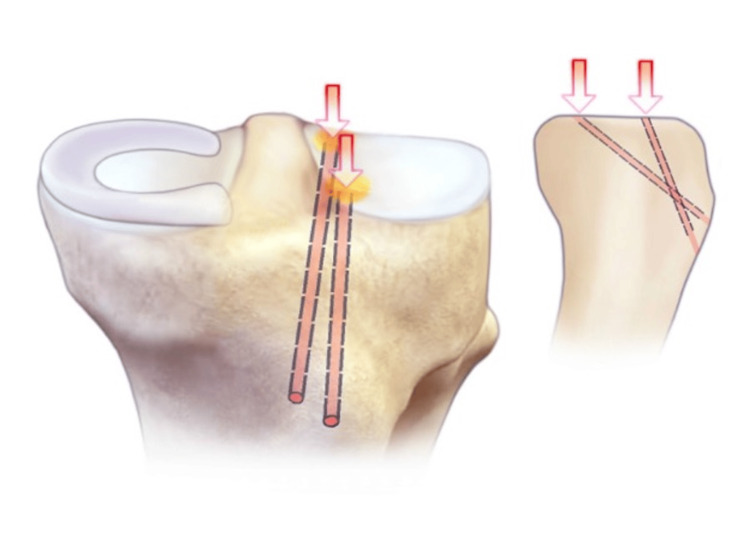
ACL guides are used to drill thin bone tunnels with an ACL flip cutter at both the anterior and posterior horn attachments of the lateral menisci. ACL: anterior cruciate ligament.

**Figure 6:**
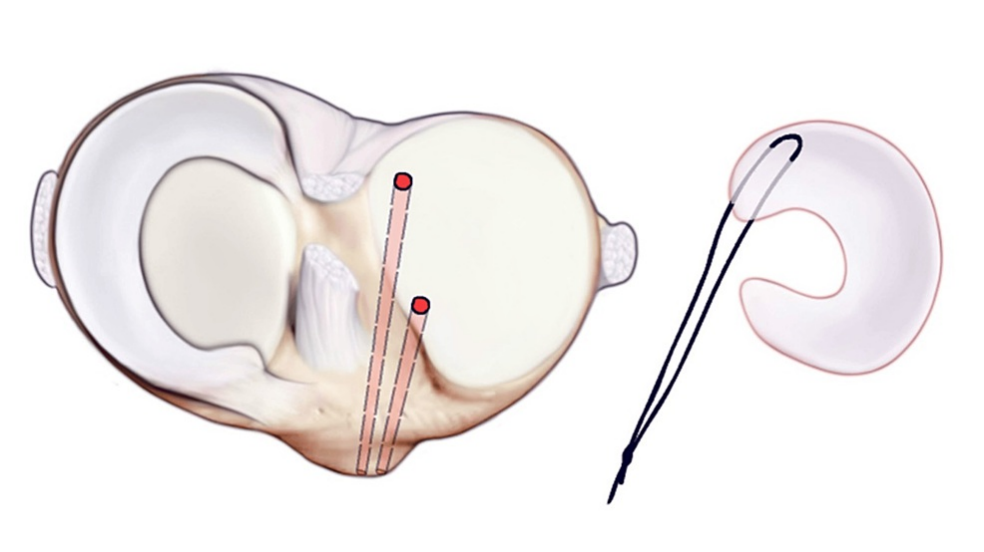
Security loops are placed using fiber loops into the anterior and posterior horns of the meniscus allograph. A dynacord passing stitch is then placed through the center of both anterior and posterior horn attachments.

**Figure 7:**
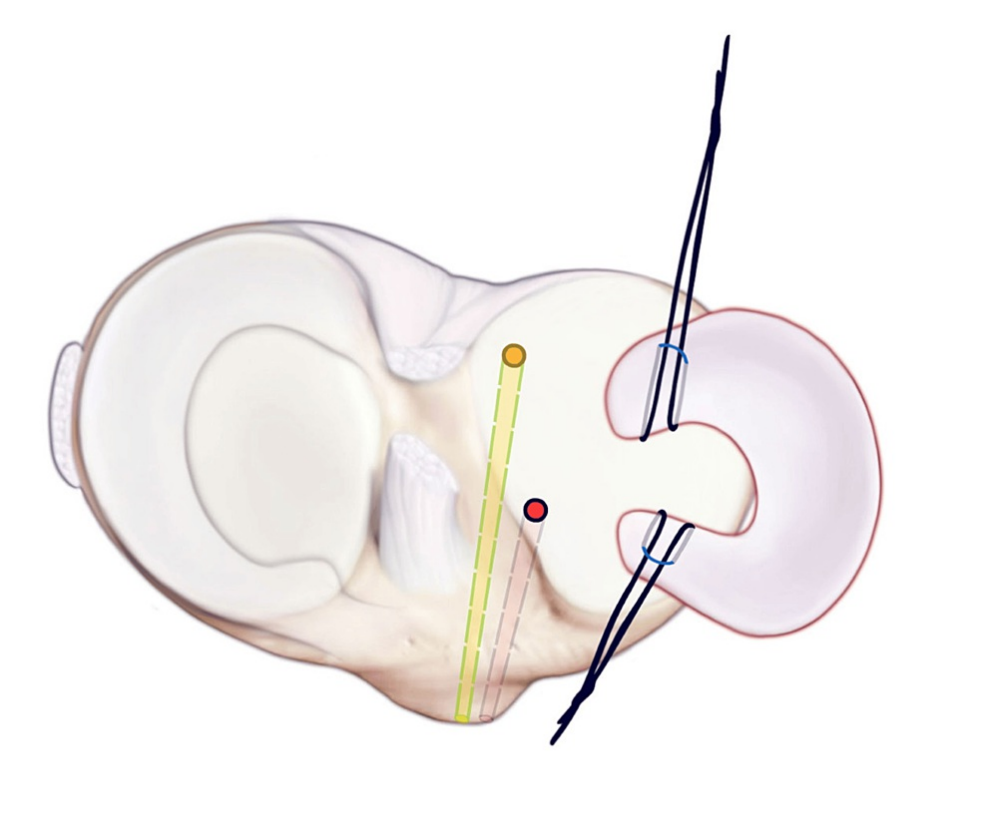
The posterior security loop is shuttled through the posterior tunnel. Next, a number two fiber wire suture is run through the posterior and anterior horns of the size-matched allograft and secured via horizontal mattress configurations.

**Figure 8:**
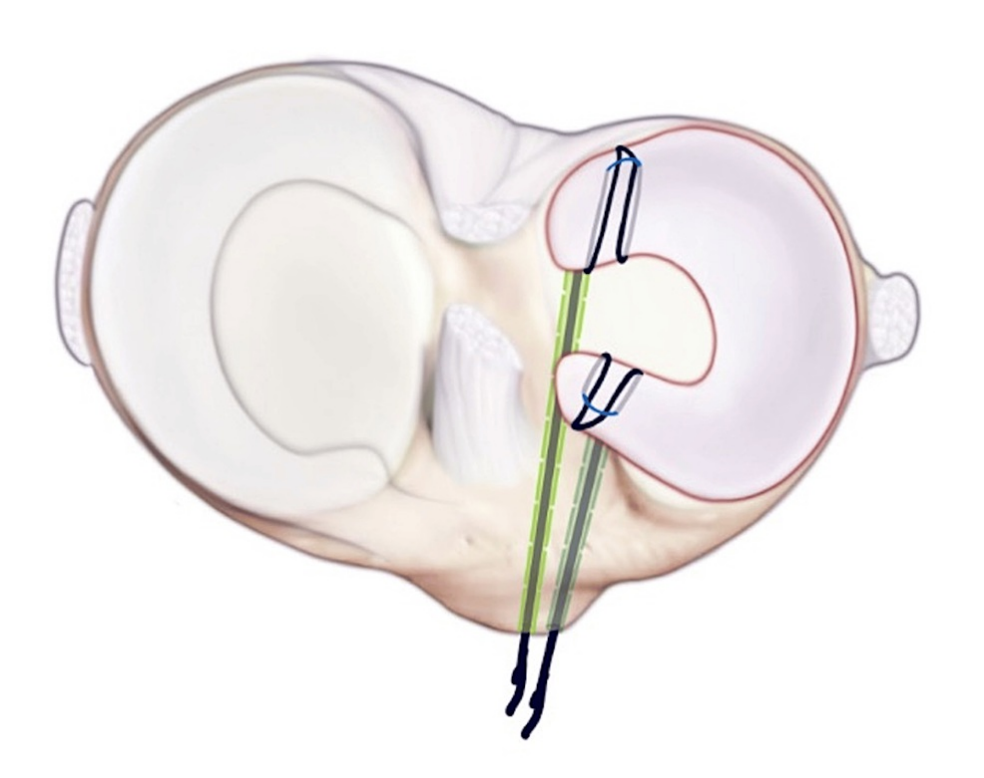
The meniscus is shuttled into the knee, and the anterior horn of the meniscus is shuttled, and then a number two fiber wire suture is run through the anterior and posterior horns of the allograft, which is secured via horizontal mattress configurations.

**Figure 9:**
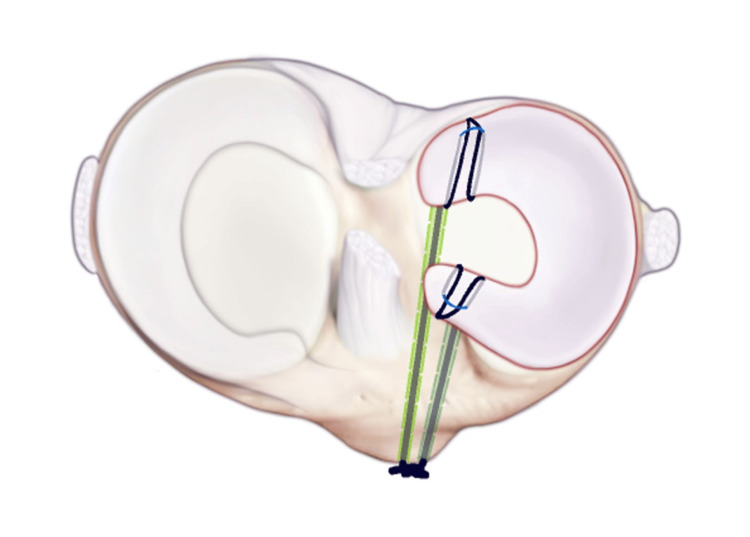
The fiber loops on the outside cortex for cortical fixation. The allograft is then shuttled using a dynacord passing stitch over the tibia and tied over a bone bridge through a small anteriorly placed access incision.

**Figure 10:**
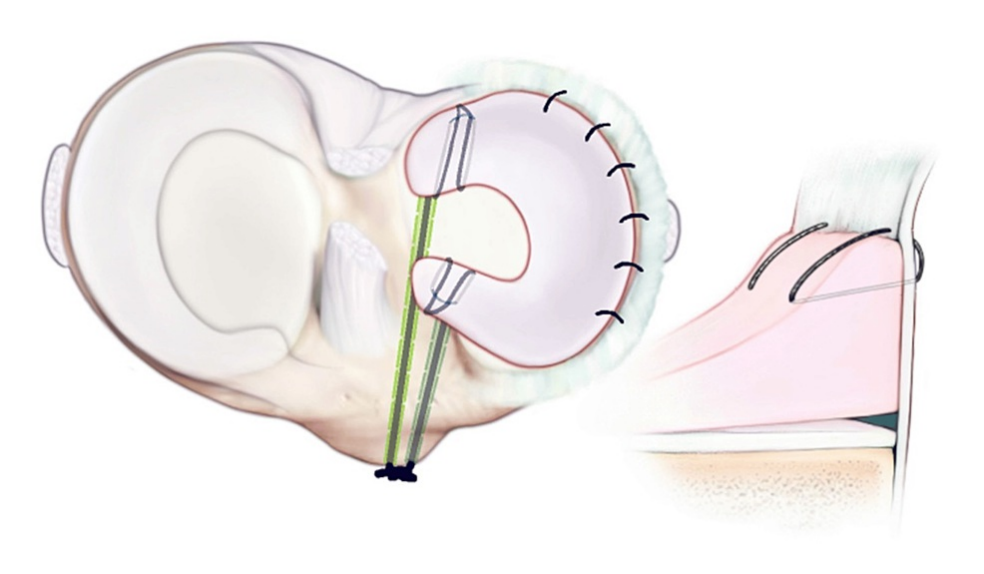
The meniscus is repaired circumferentially with vertical mattress sutures. The allograft is then secured peripherally with a variety of meniscal repair techniques. The body of the meniscus is first secured with an all-inside technique using two vertical mattress 2-span meniscus sutures. The graft is secured anteriorly with an outside-in technique using a spinal needle and #0 Proline. Posteriorly, a mini-open technique is utilized. A mini-open incision is made posterolaterally. A retractor is placed between the neurovascular structures in the posterior capsule. Then, a 2-0 fiber wire on a flexible needle is utilized to pass two vertical mattress sutures into the posterior horn. The portal incisions are closed using simple nylon sutures. The mini-open incision and the small access incision anteriorly are closed using #0 Vicryl, 2-0 Vicryl, and running subcuticular biosyn sutures. A polar care unit is applied for postoperative pain control and swelling.

The senior author’s novel technique for lateral meniscal allograft transplantation through suture soft tissue fixation in an arthroscopic and mini-open partial inside/outside technique has demonstrated decreased blood loss, decreased surgical time, and decreased intra-operative trauma using two vertical mattress 2 span meniscus sutures. Each patient has MRIs and X-rays pre-operatively. X-rays are used to calculate the size of the graft. All allograft menisci are from RTI Surgical and are sterilized using their BioCleanse processing to remove any bacteria, viruses, fungi, and spores. As shown in Figures 3-9, the graft is secured anteriorly with an outside-in technique using a spinal needle and #0 Proline. Posteriorly, a mini-open technique is utilized. A mini-open incision is made posterolaterally. A retractor is placed between the neurovascular structures in the posterior capsule. As shown in Figure 10, a 2-0 Fiber wire on a flexible needle is utilized to pass two vertical mattress sutures into the posterior horn. Once this is tied down, this results in anatomical repair. All instruments are removed, and the knee is drained of fluid. The portal incisions are closed using simple nylon sutures. The mini-open incision and the small access incision anteriorly are closed using #0 Vicryl, 2-0 Vicryl, and running subcuticular biosyn sutures. A polar care unit is applied for postoperative pain control and swelling. The patient's knee is placed in a hinged-knee brace and locked in an extension. The postoperative course includes wearing the hinged-knee brace in extension and remaining NWB on the transplant side until six weeks postoperatively with Aspirin 325 mg per day for DVT prophylaxis. At six weeks, the patient may progress to weight-bearing as tolerated with the help of formal physical therapy. At two weeks postoperatively, the patient may begin moving the transplant site, limited to 90 degrees of flexion. 

## Conclusions

The meniscus is a vital component of the knee joint, acting as a shock absorber and aiding in the transmission of forces. Arthritic changes in the knee are common in patients who have meniscal tears or have undergone meniscectomy. Due to the importance of the meniscus in the knee joint, research has focused on treatment techniques that can spare and repair the meniscus when it is torn. However, the vasculature of the meniscus makes repair often difficult or, in some cases, impossible. A current focus in orthopedics has been on meniscal allograft transplantation to fill this gap. Surgical techniques for this procedure are constantly evolving. The lack of an accepted universal surgical technique for graft fixation, along with the current failure rates, demonstrates the need for further improvements. The senior author offers a novel technique for meniscal allograft transplantation that has shown decreased blood loss and surgical time and reduced intra-operative trauma to the knee. Our patient responded well to the surgical procedure and is thus far satisfied with the outcome. On his six-week postoperative visit, his pain was minimal, and he was walking with a normal gait.  

Although the senior author’s postoperative plan is in line with current guidelines of remaining non-weight bearing on the transplant side for six weeks, the patient had begun full weight bearing on the operative side after two weeks post-op. The maintained integrity of the graft can be appreciated despite this fast progression in weight-bearing status, showcasing the durability of this fixation technique. Of note, the senior author has performed this novel technique on six other patients with similar, successful outcomes. In the long term, we would like to continue following up with all patients to track their progress, assess for graft integrity, and ensure patient satisfaction. 
